# A Nuclear Function for Armadillo/β-Catenin

**DOI:** 10.1371/journal.pbio.0020095

**Published:** 2004-02-10

**Authors:** Nicholas S Tolwinski, Eric Wieschaus

**Affiliations:** **1**Howard Hughes Medical Institute, Department of Molecular BiologyPrinceton University, Princeton, New JerseyUnited States of America

## Abstract

The Wnt signaling pathway provides key information during development of vertebrates and invertebrates, and mutations in this pathway lead to various forms of cancer. Wnt binding to its receptor causes the stabilization and nuclear localization of β-catenin. Nuclear β-catenin then functions to activate transcription in conjunction with the transcription factor TCF. A recent report has challenged this basic precept of the Wnt signaling field, arguing that the nuclear localization of β-catenin may be unrelated to its function and that β-catenin functions at the plasma membrane to activate this signaling pathway. Here we present evidence that the pathway in fact does depend on the nuclear localization of β-catenin. We reexamine the functionality of various truncations of β-catenin and find that only the most severe truncations are true signaling-null mutations. Further, we define a signaling-null condition and use it to show that membrane-tethered β-catenin is insufficient to activate transcription. We also define two novel loss-of-function mutations that are not truncations, but are missense point mutations that retain protein stability. These alleles allow us to show that the membrane-bound form of activated β-catenin does indeed depend on the endogenous protein. Further, this activity is dependent on the presence of the C-terminus-specific negative regulator Chibby. Our data clearly show that nuclear localization of β-catenin is in fact necessary for Wnt pathway activation.

## Abstract 2

An uncertainty about Wnt signalling -- and in particular about the role of beta-catenin -- is laid to rest

## Introduction

The Wnt signal transduction pathway has been studied extensively in both vertebrate and invertebrate systems. The *Drosophila* ortholog *wingless* (*wg*) is a segment polarity gene that defines posterior cell fates in each of the larval segments (for a review of the various functions of Wg, see Wodarz and Nusse 1998). The pathway is activated when the extracellular ligand Wg binds to the transmembrane receptors Frizzled and Arrow. These in turn activate Disheveled (Dsh), which inactivates a complex composed of Axin, adenomatous polyposis coli (APC), and Zeste-white 3 (Zw3) (the *Drosophila* homolog of glycogen synthase kinase [GSK3β]). This complex is responsible for the retention of Armadillo (Arm) in the cytoplasm, for its phosphorylation, and thus for its targeting for ubiquitination and destruction. When the complex is inactivated by Dsh, the intracellular levels of Arm increase, and Arm enters the nucleus, where in combination with the transcription factor TCF/Pangolin, it activates the transcription of genes such as cyclin D and c-*myc* (Wodarz and Nusse 1998).

We have argued that Axin plays a key role in the Wnt signaling process, functioning both as an anchor for Arm and a scaffold for the degradation complex. Wnt signaling results in a visible reduction in Axin levels, and mutations in Axin cause a relocalization of Arm to the nucleus (Tolwinski and Wieschaus 2001; Tolwinski et al. 2003). The nuclear import and export of Arm are not clearly understood (for a review, see Henderson and Fagotto 2002), but Arm can cross the nuclear membrane by interacting with the nuclear pore complex directly. Once in the nucleus, Arm interacts with a variety of nuclear factors, in particular the transcription factor TCF/LEF (Behrens et al. 1996; Molenaar et al. 1996; Brunner et al. 1997; van de Wetering et al. 1997). The β-catenin–TCF complex releases repression and activates transcription (Cavallo et al. 1998).

A recent study has challenged this view and has questioned the importance of nuclear localization of Arm protein (Chan and Struhl 2002). These authors' conclusions were based primarily on the observation that a membrane-tethered, stabilized form of Arm (Arm^ΔArm^) causes activation of the Wnt pathway without entering the nucleus. However, this is not the first time that the controversy about the location of Arm/β-catenin function has arisen. Previously, a group working with amphibian embryos had found that membrane-tethered plakoglobin, a close relative of β-catenin, can activate Wnt signaling (Merriam et al. 1997). Another group showed, however, that expression of membrane-tethered forms of β-catenin leads to the nuclear localization of endogenous β-catenin (Miller and Moon 1997). When the endogenous Arm/β-catenin gene was mutated, the activity of membrane-tethered forms was lost (Cox et al. 1999b). These experiments illustrate the importance of following the activity of the endogenous allele in evaluating the activity of membrane-tethered forms. Previously, we had expressed the same membrane-tethered form used by Chan and Struhl (2002) in embryos with various endogenous *arm* mutations and had concluded that it functions by titrating Axin to the membrane, releasing the endogenous Arm protein and allowing it to move freely into the nucleus (Tolwinski and Wieschaus 2001). These experiments are difficult, because none of the cell-viable alleles are absolute genetic nulls, as Arm plays essential roles in both Wnt signaling and cell adhesion.

In this study, we reexamine Arm function using three classes of previously described *arm* alleles. We find that by manipulating their levels and localizations, many alleles believed to be signaling nulls can still activate transcription. When the cell-adhesive defects of the most severe class of alleles are rescued, however, the mutant protein still fails to signal, allowing us to assay the activity of membrane-tethered Arm in a true signaling-null background. We find that nuclear localization is necessary for pathway activation and that exclusively membrane-bound forms of Arm are insufficient for this. We use two novel missense mutations in *arm* to assess the nuclear activity of Arm and confirm that negative regulation by the transcriptional regulator Chibby (Cby) is required for patterning.

## Results

### Membrane-Tethered Arm Is Dependent upon the Endogenous *arm* Allele

**Figure 1:**
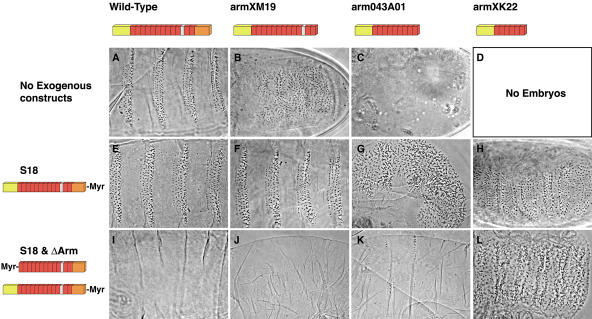
Arm^ΔArm^ Requires Endogenous Arm Endogenous allele indicated at top; ectopically expressed transgenes indicated at left. (A) The wild-type cuticle of a *Drosophila* embryo. (B) The *arm^XM19^* “weak” allele phenotype, similar to *wg* mutations in which the entire cuticle is covered with denticles. (C) The *arm^O43A01^* “medium” allele phenotype shows disintegrated embryos in which cells delaminate owing to an inability to form adherens junctions. (D) *arm^XK22^* “strong” allele does not produce embryos, owing to an oogenesis defect. (E) A wild-type embryo expressing Arm^S18^ shows a wild-type cuticle. (F) *arm^XM19^* mutant expressing Arm^S18^ is rescued to a wild-type cuticle. (G) *arm^O43A01^* mutant expressing Arm^S18^ shows rescued adhesion, but a *wg* mutant signaling phenotype. (H) *arm^XK22^* mutant expressing Arm^S18^ also shows rescued adhesion, as well as a *wg* mutant signaling phenotype. (I) Coexpression of Arm^ΔArm^ and Arm^S18^ in wild-type embryos leads to naked cuticle or the uniform Wg active phenotype. (J) Coexpression of Arm^ΔArm^ and Arm^S18^ leads to naked cuticle or the uniform Wg active phenotype in an *arm^XM19^* mutant background. (K) Coexpression of Arm^ΔArm^ and Arm^S18^ in *arm^O43A01^* mutant embryos leads to naked cuticle or the uniform Wg active phenotype. (L) However, coexpression of Arm^ΔArm^ and Arm^S18^ in “strong” mutant *arm^XK22^* background shifts embryos back to the *wg* mutant phenotype. Expression of the membrane-tethered, stabilized form of Arm (Arm^ΔArm^) leads to uniform activation of signaling in all cells. This effect is independent of whether the cell is exposed to Wg signal or not, because Arm^ΔArm^ functions independently of Wg ligand. The membrane-tethered, unstabilized form of Arm (Arm^S18^) leads to pathway activation only in cells that receive Wg signal, because this form of Arm is still subject to Wg-dependent phosphorylation and phosphorylation-dependent degradation.

**Figure 2:**
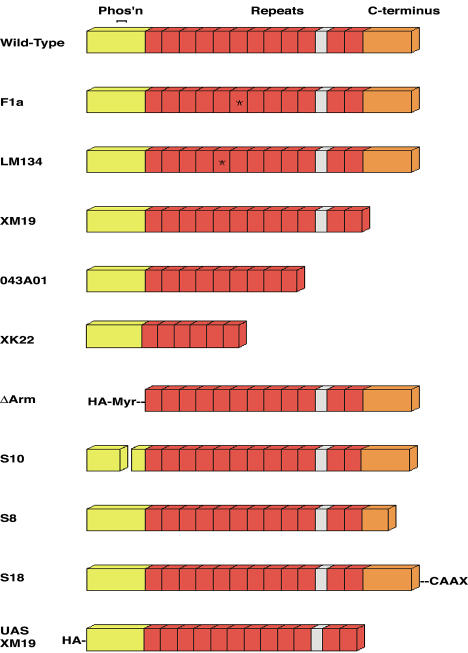
Structure of Arm Protein and Alleles Arm protein consists of three regions. The N-terminus is required for transactivation, for phosphorylation-based and proteasome-mediated degradation, and for α-catenin binding. The central repeats region is a superhelical structure that contains the binding sites for most of Arm's binding partners, including APC, TCF, Cadherin, and Axin. The C-terminus is required for Cby and Teashirt binding and transactivation. The *arm^F1a^* mutation causes an arginine-to-histidine change within repeat six. The *arm^LM134^* mutation causes a serine-to-phenylalanine change in repeat five. The “weak” allele *arm^XM19^* removes the entire C-terminus. The “medium” allele *arm^O43A01^* causes early termination within repeat nine. The “strong” allele *arm^XK22^* causes early termination within repeat six. The Arm^ΔArm^ transgene consists of the entire repeats region and C-terminus fused to an HA tag and myristoylation sequence at the N-terminus under GAL4/UAS control. The Arm^S10^ transgene contains a small deletion in the N-terminus, which removes the four phosphorylation sites necessary for degradation and is under GAL4/UAS control. The Arm^S8^ transgene contains a deletion of approximately a third of the C-terminus and is under endogenous promoter control. The Arm^S18^ transgene contains the entire Arm sequence fused to the CAAX myristoylation sequence of Ras and is under endogenous promoter control. The UAS–Arm^XM19^ is the equivalent of the *arm^XM19^* allele in deletion, but is fused to an N-terminal HA tag and is under GAL4/UAS control.

The original mutants in the *arm* gene were classified into three groups based upon their phenotypes and the position of stop codons that result in truncated proteins. The “weak” class has the smallest truncations and is represented by *arm^XM19^*. In germline clones (where maternal and zygotic contribution of protein is removed; Chou and Perrimon 1992), its phenotype is identical to loss-of-function *wg* mutations (Figure 1B; Peifer and Wieschaus 1990). The “medium” class, represented here by *arm^O43A01^*, shows defects in adhesion as well as transcription. Here germline clones give embryos that fail to differentiate an intact cuticle (Figure 1C; Tolwinski and Wieschaus 2001). The “strong” class (*arm^XK22^*) does not allow proper progression through oogenesis and germline clones do not make eggs (Figure 1D; Peifer et al. 1993). Cox et al. (1999b) showed that the junctional defects of the “medium” alleles can be circumvented by coexpression of a membrane-tethered full-length form of Arm (Arm^S18^) (Figure 2). We have confirmed their findings and extended them to the “strong” allele during oogenesis. We show that uniform expression of Arm^S18^ allows *arm^XK22^*germ cells to produce normal eggs and rescues the adhesive defects of both *arm^XK22^* and *arm^O43A01^* embryos. The membrane-tethered form does not, however, rescue the signaling defects associated with either of these alleles and the embryos show typical *wg* phenotypes (see Figure 1G and 1H).

**Figure 3:**
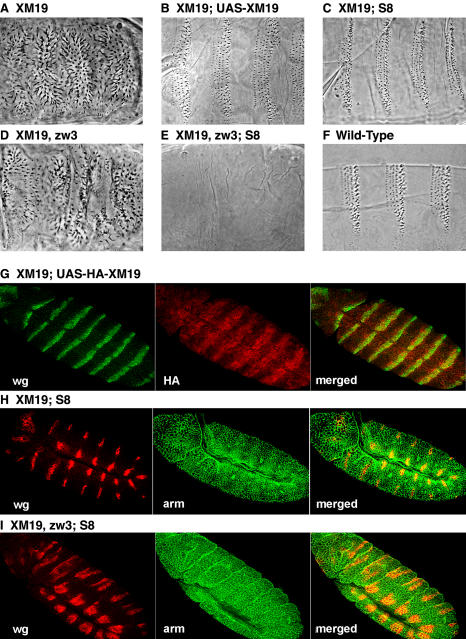
C-Terminally Truncated Arm Can Signal If Its Levels Are Increased (A) *arm^XM19^* shows a *wg* mutant phenotype. (B) Expression of GAL4/UAS-driven Arm^XM19^ protein in *arm^XM19^* mutant background rescues this to a wild-type pattern. (C) The same is true of expression of an endogenous promoter-driven truncation Arm^S8^. (D) Removal of Zw3 has no effect on *arm^XM19^* cuticle pattern. (E) However, when Arm^S8^ is introduced into *arm^XM19^, zw3* mutants, the cuticle is naked. (F) Wild-type embryo is shown for comparison. (G–I) Arm stainings reveal that expression of UAS–Arm^XM19^ (stained for the HA tag [G]) and Arm^S8^ (stained for Arm [H]) is present in stripes corresponding to Wg striping, whereas removal of Zw3, along with Arm^S8^ expression, leads to uniform and high levels of Arm throughout the epidermis (I).

Expression of Arm^S18^ has no effect on the cuticle of wild-type embryos (compare Figure 1E to 1A), but it does rescue the signaling defects of *arm* alleles, like *arm^XM19^*, that have only short C-terminal truncations (Cox et al. 1999b; see Figure 1F). These alleles normally show very low levels of protein (Peifer and Wieschaus 1990), and Cox et al. (1999b) postulated that expression of a membrane-tethered Arm might “free up” the endogenous mutant protein, allowing the “weak” allele to signal. The low levels of *arm^XM19^* may reflect degradation of nonsense mRNAs triggered by the premature stop codon in this mutant (reviewed in Wagner and Lykke-Andersen 2002). To eliminate this degradation, we expressed a cDNA version of the *arm^XM19^* allele under GAL4/UAS control (Brand and Perrimon 1993) in embryos mutant for *arm^XM19^* (Figure 3B). To avoid the possibility of overexpression artifacts, we also expressed a smaller C-terminal deletion from the endogenous promoter (Arm^S8^; Orsulic and Peifer 1996). In both experiments, the truncated protein from the transgene accumulated to levels approximating those observed in wild-type (Figure 3G and 3H) and in the characteristic striped pattern indicative of response to the Wg signal (Peifer and Wieschaus 1990). The truncated protein rescued the *arm^XM19^* phenotype to a wild-type cuticle pattern and allowed hatching (Figure 3C). When combined with a mutation in the kinase *zw3*, Arm^S8^ causes the cuticles of these embryos to appear uniformly naked (compare Figure 3E to 3D), as would be expected since the Arm^S8^ protein is expressed to high uniform levels throughout the epidermis when Zw3 is removed (Figure 3I). These experiments argue that the C-terminus is not essential for signaling or transcriptional activation of Wnt targets required for cuticle patterning. However, as we do not obtain adult flies containing exclusively the truncated alleles, it is very likely that the C-terminus is not entirely expendable and must have important functions later in development.

### Null Allele Background Proves That Arm^ΔArm^ Cannot Signal on Its Own

The fact that *arm^XM19^* is able to signal when expressed at normal levels invalidates its use in tests for a direct activity of membrane-tethered Arm in Wnt signaling (Chan and Struhl 2002). Therefore, expression of Arm^ΔArm^ in a “weak” allele background cannot address whether membrane-tethered Arm activates transcription without ever entering the nucleus, since a membrane-untethered, signaling-competent form of Arm is also present. To directly address whether the Arm^ΔArm^ transgene can transmit Wg signal on its own, we turned to the “strong” and “medium” alleles. Although Arm^S18^ is not sufficient to restore signaling to these alleles, it raises the possibility that stronger expression of stabilized, membrane-tethered Arm (Arm^ΔArm^) might reveal some signaling capacity of those alleles as well. Experiments of this kind have been difficult with Arm^ΔArm^, given that it lacks the α-catenin-binding site and fails to rescue the junctional defect in “medium” and “strong” endogenous *arm* allele backgrounds. We have found that by expressing both Arm^ΔArm^ and Arm^S18^, we can recover intact embryos in all backgrounds tested. We find that “medium” and “weak” alleles can be induced to activate transcription, but the “strong” *arm* allele cannot (see Figure 1J–1L), consistent with the position of the “medium” alleles in the hypomorphic allelic series. These findings demonstrate that Arm^ΔArm^ is dependent upon the endogenous form of *arm*, as it cannot activate transcription in the “strong” allele background.

### Loss-of-Function Missense Mutations

**Figure 4:**
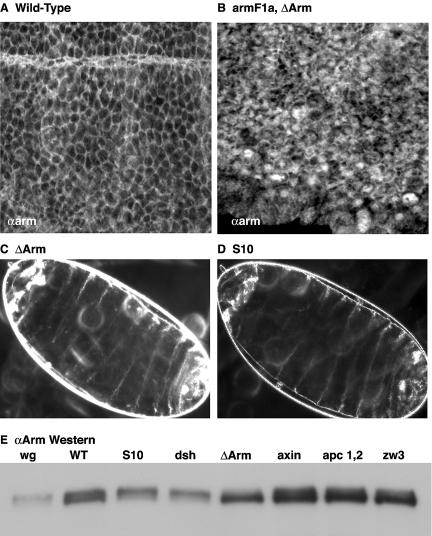
Expression of Arm^ΔArm^ Leads to the Nuclear Localization of Endogenous Arm Protein (A) Wild-type Arm protein appears in stripes that correspond to cells responding to Wg signaling. (B) Expression of Arm^ΔArm^ in an *arm^F1a^* background leads to the nuclear localization of endogenous Arm. (C and D) Dark-field images reveal that expression of both Arm^ΔArm^ and Arm^S10^ leads to similar naked cuticle phenotypes. (E) An anti-Arm Western blot showing a faster-migrating band, which correlates with endogenous Arm's being active, and a slower-migrating band, which correlates with Arm's being inactive.

**Figure 5:**
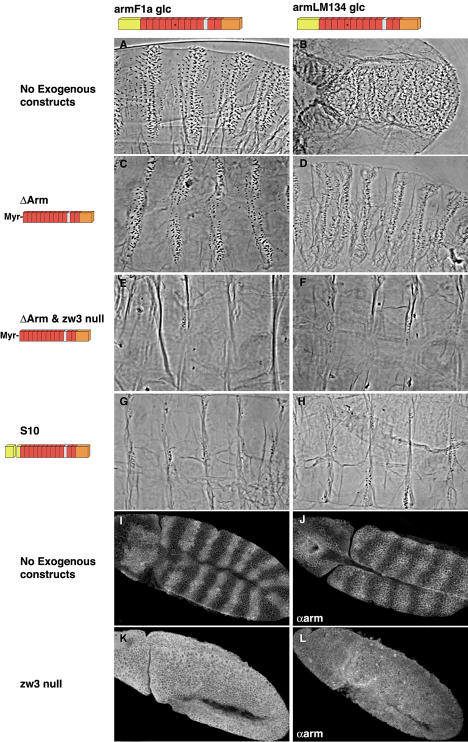
ΔArm Functions through Endogenous Arm (A) Embryonic cuticle of *arm^F1a^* mutant showing a weak loss-of-function phenotype. (B) Cuticle of *arm^LM134^* mutant embryo showing a strong loss-of-signaling phenotype. (C) Embryo mutant for *arm^F1a^* expressing Arm^ΔArm^ showing relatively normal segment polarity. (D) *arm^LM134^* mutant expressing Arm^ΔArm^ also shows segment polarity. (E and F) Both alleles in combination with a null *zw3* allele and expressing Arm^ΔArm^ show a complete lack of denticles. (G and H) Both alleles expressing the activated but nontethered form of stabilized Arm, Arm^S10^, show the naked cuticle phenotype. (I–L) In both missense alleles, the mutant protein is expressed in stripes (I and J), corresponding with Wg expression (data not shown), which is abolished when the key degradation kinase Zw3 is removed (K and L).

When Arm^ΔArm^ is expressed in a wild-type embryo, it strongly activates Wg signaling (Figure 4C; Chan and Struhl 2002). Chan and Struhl (2002) suggest that this is because this membrane-tethered form of Arm can signal on its own. The results presented above argue, on the other hand, that it does so by stabilizing the endogenous protein. To further test this, we asked whether expression of Arm^ΔArm^ can induce Wg signaling when endogenous Arm is replaced by signaling-deficient Arm. We turned to two novel missense mutations where the rest of the *arm* coding region remains intact. Because these alleles do not produce truncations through stop codons, they are immune to nonsense mRNA degradation (Wagner and Lykke-Andersen 2002). Both mutations result in amino acid substitutions close to repeat seven, a key hinge region postulated to be important in binding of TCF (Huber et al. 1997; Graham et al. 2000). Both mutants retain the phosphorylation sites required for degradation and therefore accumulate in stripes in response to Wg signal (Figure 5I and 5J). They supply apparent wild-type junctional activity and accumulate to high levels in all cells when the kinase responsible for the degradation signal (Zw3) is removed (Figure 5K and 5L). The primary phenotype of these alleles is a loss or reduction of Wnt transcriptional responses (Figure 5A and 5B). The *arm^F1a^* allele produced a partial loss-of-function phenotype, and germline clone embryos show some residual naked cuticle. *arm^LM134^* produces a stronger phenotype comparable to a loss of *wg* function, although it may not be a signaling null (see below).

We asked whether these signaling-deficient alleles could block the cell fate transformation and Wnt target activation observed when Arm^ΔArm^ is expressed in wild-type epidermis. If Arm^ΔArm^ functions independently of the endogenous protein, then all cells should assume the naked cell fate. However, this does not occur (Figure 5C and 5D). Instead, both point mutants produce a cuticle pattern with periodic denticle belts and regions of intervening naked cuticle. This periodicity may reflect the fact that *arm^F1a^* and *arm^LM134^* can still be controlled by Wg even when Arm^ΔArm^ is expressed. This periodicity is, in fact, abolished when Zw3 activity is removed from such embryos (i.e., in triply mutant *zw3, arm^F1a^;*Arm^ΔArm^ embryos). Under these conditions, all cells in the cuticle take on the naked cell fate (Figure 5E and 5F). Since Arm^ΔArm^ lacks the N-terminal sites that respond to Zw3, the sensitivity of the double-mutant phenotype confirms that the pattern of the double mutant is dependent on the endogenous Arm protein.

The behavior of membrane-tethered Arm^ΔArm^ contrasts with that of other stabilized forms of Arm that would be predicted to move more freely between the cytoplasm and the nucleus. Arm^S10^, for example, contains a small N-terminal deletion that blocks Zw3 phosphorylation, but preserves binding sites for various nuclear proteins (see Figure 2; Pai et al. 1997). Arm^S10^ is not membrane-tethered, but the cell fate transformations it produces are identical to those produced by Arm^ΔArm^ (compare Figure 4C and 4D). They do not, however, depend on the endogenous allele and are still observed in an *arm^F1a^* or *arm^LM134^* germline clone background (Figure 5G and 5H).

### Arm^ΔArm^ Causes Nuclear Localization and Mobility Shift of Endogenous Arm

All of our experiments argue that Arm^ΔArm^ produces its effect on transcription by activating the endogenous alleles. To investigate the mechanism that underlies this effect, we looked at the in situ localization of the endogenous Arm protein and its migration pattern on Western blots. Expression of Arm^ΔArm^ is sufficient to drive both wild-type and the point mutant forms of Arm into nuclei (see Figure 4A and 4B; Miller and Moon 1997; Tolwinski and Wieschaus 2001).

Generally, the most obvious feature observed upon removal of any of the negative factors of the Wg pathway is the rapid accumulation of Arm in cells. However, another feature is the phosphorylation state of the Arm protein. Peifer et al. (1994a) found that a fast-migrating band of Arm corresponds with active Wg signaling and that a slower-migrating band corresponds with Wg's being off. Therefore, it is the unphosphorylated band that corresponds with signaling. Here we show that, on Western blots, endogenous Arm protein responds to Arm^ΔArm^ expression in much the same way that it does to the removal of negative components of the pathway such as Axin and APC1 and APC2 (see Figure 4E). We see a downshift of the protein, which is directly opposite to what is seen when a positive component of the pathway is removed (Dsh or Wg; see Figure 4E). Wild-type embryos show the expected intermediate phenotype, as they have both active and inactive forms of Arm protein (see Figure 4E). The observed shift is most likely the result of phosphorylation (Peifer et al. 1994a), though we do not address this directly in this study.

### The C-Terminus of Arm Is Necessary for Cby-Mediated Repression

**Figure 6:**
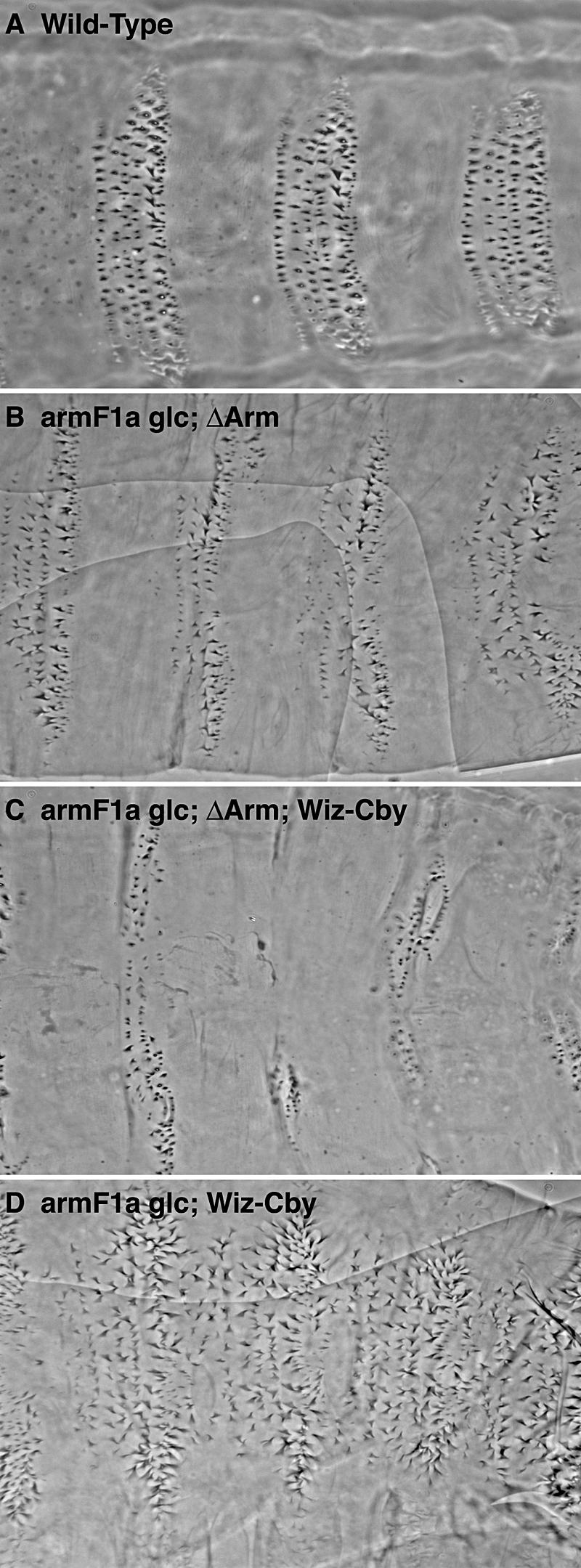
Relief of C-Terminal Repression through the Elimination of Cby Leads to Uniform Activation of Signaling (A) A wild-type cuticle shown for comparison. (B) Expression of Arm^ΔArm^ in the *arm^F1a^* background. (C) Expression of a Cby RNAi construct along with Arm^ΔArm^ in the *arm^F1a^* background. (D) Expression of a Cby RNAi construct in an *arm^F1a^* background.

Although the missense mutations we have used in our studies produce (on average) weaker phenotypes, they are more effective at blocking the cell-fate transformation induced by Arm^ΔArm^ than the “medium” C-terminal truncation mutants (compare Figure 1K with Figure 5C and 5D). The comparison is somewhat indirect, owing to the necessity of expressing Arm^S18^ in the “medium” *arm* allele background in order to get intact embryos. However, we find that expression of Arm^S18^ in an *arm^F1a^* background has no visible effect on the cuticle (data not shown). Therefore, the activity of C-terminally truncated *arm* alleles in response to ΔArm expression suggests that, under certain conditions, removal of the C-terminus may actually enhance the transcriptional activity of Arm. One possibility is suggested by the recent discovery of Cby (Takemaru et al. 2003), a nuclear negative regulator of the Wg pathway that binds to the C-terminus of Arm. To test whether nuclear Cby affected the transformation produced by Arm^ΔArm^, we used RNA interference (RNAi) to reduce Cby levels in *arm^F1a^* embryos with and without Arm^ΔArm^. In the absence of Arm^ΔArm^, i.e., in embryos where most Arm^F1a^ protein is cytoplasmic, Cby RNAi has no effect (Figure 6D). However, when Arm^ΔArm^ is present, lowering Cby levels leads to increased naked cuticle characteristic of Wnt pathway activation (compare Figure 6B to 6C). We propose that Cby's effect on *arm^F1a^* protein is dependent on Arm^ΔArm^ relocalizing Arm to the nucleus.

## Discussion

In this study we offer genetic proof that the nuclear localization of Arm is important for the activation of the pathway. The dissenting view (Chan and Struhl 2002) relied on C-terminal truncations that we have shown retain their ability to signal if their levels are increased. These alleles also appear to bypass the normal nuclear regulation by Cby. We show that full-length loss-of-function forms of Arm provide a novel way of assessing the activity of the pathway. Finally, we show that in an approximate signaling-null condition, Arm^ΔArm^ cannot activate transcription on its own. Based on these findings, we propose that membrane-tethered Arm, whether wild-type or activated, cannot activate transcription on its own. It does, however, have a profound effect on the endogenous form, forcing both “weak” and “medium” alleles to translocate to the nucleus and activate transcription. Our findings extend and build upon the original nuclear localization of Arm model (Miller and Moon 1997; Cox et al. 1999b). Further support for the nuclear localization of Arm model has recently been provided by the publication of a study that uses tissue culture experiments to show that nuclear localization of Arm is required (Cong et al. 2003).

Our results also point to an unexpected feature of Arm, namely that the C-terminus, although it has been shown to supply transcriptional activation (Hsu et al. 1998), does not appear to be required for Wnt activation. Cox et al. (1999a) studied this aspect of Arm function and found that a C-terminally truncated form of Arm can significantly rescue the signaling defects of *arm* mutants, but is not as good as the wild-type form at transcriptional activation. Further, given that *arm* mutant flies expressing the transgene that lacks the C-terminus do not survive to adulthood, the C-terminus may not be entirely expendable. This may point to the requirement for Cby-based repression or Teashirt-mediated activation at a later stage of development, as both these proteins function by binding the C-terminus of Arm (Gallet et al. 1999; Takemaru et al. 2003). However, taken together with the finding that an N-terminally truncated Arm sent to the nucleus fails to activate transcription (Chan and Struhl 2002), it appears that it is the N-terminus that is most important for the nuclear transactivation and chromatin remodeling functions ascribed to β-catenin (Hsu et al. 1998; Hecht and Kemler 2000; Takemaru and Moon 2000; Barker et al. 2001; Tutter et al. 2001; Bienz and Clevers 2003).

We have previously shown that the “medium” *arm* mutant (*arm^O43A01^*, which creates a stop codon eliminating repeats 10 through 12 and the entire C-terminus) does not signal in the presence of uniform Arm^ΔArm^ (Tolwinski and Wieschaus 2001). Chan and Struhl (2002) found that *arm^O43A01^* embryos expressing high levels of Arm^ΔArm^ from the paired GAL4 driver were able to activate Wnt targets. But since neither Arm^ΔArm^ nor *arm^O43A01^* can provide junctional Arm activity, the abnormalities of these embryos make these experiments difficult to interpret. As an alternative, we used a membrane-tethered but otherwise wild-type form of Arm (Arm^S18^), which we expressed in *arm^O43A01^* mutant embryos (see Figure 1G). The Arm^S18^ allele rescues the junctional defects, but does not allow signaling. Similar results have been obtained with another “medium” allele, *arm^XP33^* (Cox et al. 1999b). However, when combined with Arm^ΔArm^ and Arm^S18^, *arm^O43A01^* can now be clearly seen to activate naked cell fates. It thus appears that even the “medium” alleles of *arm* actually do retain some ability to function when Arm^ΔArm^ is present. This is not observed in the larger truncations (“strong” alleles), consistent with the “medium” alleles retaining the TCF-binding region (Graham et al. 2000).

The question now becomes what is Arm^ΔArm^ doing at the membrane that causes such drastic change in the signaling kinetics of the pathway. We have previously argued that Arm^ΔArm^ may function by titrating the cytoplasmic anchoring activity of Axin and by therefore allowing rapid enrichment of Arm in the nucleus. We have in fact observed such an enrichment and have shown that it is counteracted by increasing the level of Axin (Tolwinski and Wieschaus 2001). Further work has pointed to the importance of controlling Axin stability in pathway activation (Salic et al. 2000; Mao et al. 2001; Lee et al. 2003; Tolwinski et al. 2003). Expression of large quantities of a stabilized, membrane-tethered form of Arm might also remove additional cytoplasmic inhibitory factors, preventing them from interacting with nontethered Arm. In turn, even lower-level or lower-activity alleles will now be able to activate transcription, simply owing to the complete lack of inhibiting factors.

The missense mutations described here provide a glimpse of the in vivo activity of Arm protein. Structural studies of β-catenin found that although the central repeat region forms a uniformly repeating super helix, one α-helix was missing from repeat seven. The missing helix might allow a local flexibility in the structure and led the authors to define this region as a potential hinge (Huber et al. 1997). Further crystallographic analysis concluded that this region was important for TCF binding (Graham et al. 2000). Both our point mutations cluster around this repeat and would probably lead to structural consequences for this hinge. The apparent specificity of these alleles for the transcriptional response to Wnt signaling provides in vivo evidence that the postulated hinge may be very important for that aspect of Arm protein function.

## Note 

As the final version of this paper was being prepared, the paper by Chan and Struhl (2002) was retracted.

## Materials and Methods

### Fly Strains

The wild-type strain used was Oregon R. See Flybase (http://flybase.bio.indiana.edu) for details on mutants used. Hypomorphic mutants of *arm* are as follows: *arm^LM134^* TCC to TTC at nucleotide 2776, *arm^F1a^* CGC to CAC at nucleotide 2990, *arm^XM19^* stop codon at nucleotide 3850, *arm^O43A01^* stop codon at nucleotide 3404, *arm^XP33^* stop codon at nucleotide 3466, *arm^XK22^* stop codon at nucleotide 3013. Other alleles used were *axin^S044230^* , *zw3^M11–1^*, *dsh^V26^*, *apc1^Q8^*, *apc2^d40^*, and *wg^IG22^*.

### Crosses and Expression of UAS Constructs

#### *arm* mutants

As Arm and many other *Drosophila* proteins are contributed maternally, to fully evaluate the function of a mutant protein, one needs to make embryos maternally and zygotically mutant. Therefore, maternally mutant eggs were generated by the dominant female sterile technique (Chou and Perrimon 1992). For all expression experiments, the Arm–GAL4 driver was used. All X-chromosome mutants use FRT 101. The *arm* mutants used were as follows:

*arm^F1a^ zw3^M11–1^* (maternal)/Y (zygotic)

*arm^LM134^ zw3^M11–1^* (maternal)/Y (zygotic)

*arm^F1a^* (maternal)/Y (zygotic)

*arm^LM134^* (maternal)/Y (zygotic)

*arm^F1a^* (maternal)/Y (zygotic); Arm–GAL4/UAS–ΔArm (zygotic)

*arm^F1a^ zw3^M11–1^* (maternal)/Y (zygotic); Arm–GAL4/UAS–ΔArm (zygotic)

*arm^LM134^* (maternal)/Y (zygotic); Arm–GAL4/UAS–ΔArm (zygotic)

*arm^LM134^ zw3^M11–1^* (maternal)/Y (zygotic); Arm–GAL4/UAS–ΔArm (zygotic)

*arm^O43A01^* (maternal)/Y (zygotic)

*arm^O43A01^* (maternal)/Y (zygotic); Arm^S18^ (zygotic)

*arm^F1a^* (maternal)/Y (zygotic); Arm–GAL4/UAS–WIZ–Cby, UAS–ΔArm (zygotic)

*arm^F1a^* (maternal)/Y (zygotic); Arm–GAL4/UAS–WIZ–Cby (zygotic)

*arm^XK22^* (maternal)/Y (zygotic); Arm^S18^ (maternal)

*arm^XK22^* (maternal)/Y (zygotic); MAT–GAL4/UAS–ΔArm (zygotic); Arm^S18^ (maternal)

#### UAS transgenes and GAL4 driver lines

Previously published transgenes used were UAS–Arm*[S10], a small deletion of the phosphorylation sites (Pai et al. 1996); the Arm–GAL4 driver (Sanson et al. 1996); Arm^S18^, a mirystoylated, membrane-tethered full-length Arm (Cox et al. 1999b); and UAS–WIZ–Cby for RNAi (Takemaru et al. 2003). ΔArm is a pUAST transgene that deletes the first 128 amino acids, including the GSK3β and CKII phosphorylation sites, the CBP acetylation site, the α-catenin-binding domain, and a transactivation domain (Zecca et al. 1996).

### Antibodies and Immunofluorescence 

Embryos were treated and stained as described previously (Tolwinski and Wieschaus 2001), except that they were fixed with heptane/4% formaldehyde in phosphate buffer (0.1 M NaPO_4_ [pH 7.4]). The antibodies used were anti-Arm (monoclonal antibody [mAb] N2 7A1; Developmental Studies Hybridoma Bank, University of Iowa, Iowa City, Iowa, United States), rabbit anti-Arm (Peifer et al. 1994b), rabbit anti-c-Myc (Santa Cruz Biotechnology, Santa Cruz, California, United States), and anti-Sexlethal (mAb M-14; Developmental Studies Hybridoma Bank). Staining, detection, and image processing were as described previously (Tolwinski and Wieschaus 2001). Though not shown, the Sexlethal antibody was used to sex embryos. This allows for the identification of male embryos laid by germline clone mothers, which are hemizygous and therefore maternally and zygotically mutant for X-chromosome genes.

### Western Blotting

Embryos were lysed in extract buffer (50 mM Tris [pH 7.5], 150 mM NaCl, 1% NP-40, 1 mM EDTA, 10% glycerol; Complete Mini Protease, Sigma, St. Louis, Missouri, United States), and the extracts were separated by 7.5% SDS-PAGE and were blotted as described elsewhere (Peifer et al. 1994a). Maternally and zygotically mutant embryos were hand-selected using standard GFP balancers (http://flybase.bio.indiana.edu).

## Supporting Information

**Accession Numbers**

The GenBank (http://www.ncbi.nlm.nih.gov/Genbank/) accession numbers for the genes and alleles discussed in this paper are *apc1^Q8^* (U77947), *apc2^d40^* (AF091430), *arm* (X54468), *axin^S044230^* (AF086811), *dsh^V26^* (U02491), *wg^IG22^* (NM 164746), and *zw3^M11–1^* (X54005).

## Abbreviations

APC: adenomatous polyposis coli
Arm: Armadillo
Cby: Chibby
Dsh: Disheveled
GSK3β: glycogen synthase kinase 3β
mAb: monoclonal antibody
RNAi: RNA interference
Wg: Wingless
Zw3: Zeste-white 3
